# Phosphorylation of CRMP2 by Cdk5 Regulates Dendritic Spine Development of Cortical Neuron in the Mouse Hippocampus

**DOI:** 10.1155/2016/6790743

**Published:** 2015-12-27

**Authors:** Xiaohua Jin, Kodai Sasamoto, Jun Nagai, Yuki Yamazaki, Kenta Saito, Yoshio Goshima, Takafumi Inoue, Toshio Ohshima

**Affiliations:** ^1^Laboratory for Molecular Brain Science, Department of Life Science and Medical Bioscience, Waseda University, Tokyo 162-8480, Japan; ^2^Laboratory for Neurophysiology, Department of Life Science and Medical Bioscience, Waseda University, Tokyo 162-8480, Japan; ^3^Department of Molecular Pharmacology and Neurobiology, Graduate School of Medicine, Yokohama City University, Yokohama 236-0004, Japan

## Abstract

Proper density and morphology of dendritic spines are important for higher brain functions such as learning and memory. However, our knowledge about molecular mechanisms that regulate thedevelopment and maintenance of dendritic spines is limited. We recently reported that cyclin-dependent kinase 5 (Cdk5) is required for the development and maintenance of dendritic spines of cortical neurons in the mouse brain. Previous *in vitro* studies have suggested the involvement of Cdk5 substrates in the formation of dendritic spines; however, their role in spine development has not been tested *in vivo*. Here, we demonstrate that Cdk5 phosphorylates collapsin response mediator protein 2 (CRMP2) in the dendritic spines of cultured hippocampal neurons and *in vivo* in the mouse brain. When we eliminated CRMP2 phosphorylation in CRMP2^KI/KI^ mice, the densities of dendritic spines significantly decreased in hippocampal CA1 pyramidal neurons in the mouse brain. These results indicate that phosphorylation of CRMP2 by Cdk5 is important for dendritic spine development in cortical neurons in the mouse hippocampus.

## 1. Introduction

For the development of functional neural circuitry, the formation of synapses between appropriate partners is a critical step. The majority of excitatory synapses of postsynaptic neurons are localized in specialized cellular structures called dendritic spines. The formation, maturation, and maintenance of dendritic spines are tightly regulated by different extracellular signals including semaphorin 3A (Sema3A). Collapsin response mediator proteins (CRMPs), initially identified as a signaling molecule of Sema3A [1], are composed of five homologous cytosolic phosphoproteins (CRMP1–5) and are highly expressed in developing and adult nervous systems [2–5]. CRMPs bind with tubulin heterodimers, whereas the sequential phosphorylation of CRMPs lowers their binding affinity to tubulin [6]. CRMP2 also colocalizes with the actin cytoskeleton [7] and coimmunoprecipitates with actin [8, 9]. Phosphorylation of CRMP1 and CRMP2 by Cdk5 and sequential phosphorylation of CRMP2 by GSK-3*β* are crucial for Sema3A-induced growth cone collapse response in dorsal root ganglia (DRG) neurons [10, 11].

Recently, we demonstrated that Cdk5/p35 is necessary for dendritic spine development and maintenance [12]. Additionally, we previously showed that Sema3A-induced spine development is mediated through phosphorylation of CRMP1 by Cdk5 [13] and that CRMP1 and CRMP2 have functional redundancy in neuronal development [14]. Therefore, we hypothesized that phosphorylation of CRMP2 by Cdk5 is also important for the development of dendritic spines* in vivo*. To test this, we first analyzed the localization of phosphorylated forms of CRMP2 in the synapses of cultured hippocampal neurons and* in vivo* in the mouse hippocampus. We observed phosphorylation of CRMP2 by Cdk5 in the dendritic spines of hippocampal neurons* in vitro *and* in vivo*. We then analyzed spine densities of hippocampal CA1 pyramidal neurons in CRMP2^KI/KI^ mice in which the Cdk5 phosphorylation site of CRMP2 at amino acid 522 was changed from Ser to Ala [14]. We found reduced dendritic spine densities in hippocampal neurons in CRMP2^KI/KI^ mice. These results indicate that CRMP2 phosphorylation by Cdk5 is important for the development of dendritic spines in hippocampal neurons* in vivo.*

## 2. Materials and Methods

### 2.1. Mice

The mice used in our experiments were housed in accordance with protocols approved by the Institutional Animal Care and Use Committee at Waseda University. CRMP2^KI/KI^ mice were generated and genotyped as described previously [14]. GFP-M mice, a gift from J. Sanes [15], were crossed with these mutant mice for the present study.

### 2.2. Neuronal Culture and Immunocytochemistry

Primary cultures of hippocampal neurons were prepared from E18 Wistar rats as described previously [16], with the following modifications: cells were plated at a density of 5.0 × 10^4^ cells/well on coverslips coated with 200 *μ*g/mL poly-L-lysine (Sigma Japan, Tokyo) in 24-well plates. Neurobasal-A (Life Technologies Japan, Tokyo), B27-supplement (Miltenyi Biotec, Tokyo), 2 mM L-glutamine (Life Technologies Japan), and penicillin/streptomycin (Nacalai Tesque, Kyoto) were used as culture medium. Immunocytochemistry was performed as previously described [17]. Briefly, after washing with phosphate-buffered saline (PBS), cells were fixed with 4% paraformaldehyde (PFA) for 15 min at room temperature (RT). After washing with PBS, cells were incubated with primary antibodies, which were diluted in PBS/0.01% Triton X-100, at 4°C overnight. They were then washed 3 times with PBS and incubated with Alexa-Fluor 488 (1 : 1000) or Alexa-Fluor 568 (1 : 1000) secondary antibodies for 1 h. After 3 further washes with PBS, the sections were embedded in Vectashield mounting media (Vector Labs, Burlingame, CA). Images were obtained using a laser scanning confocal microscope based on an FV1000 scanning unit (Olympus, Japan). Primary antibodies used in this study are anti-PSD95 (mouse monoclonal, Millipore), anti-synaptophysin (mouse monoclonal, Millipore), and pCRMP2(S522), which recognizes phospho-CRMP2 at Ser522 (rabbit polyclonal, EMC Biosciences).

### 2.3. Histological Analysis

#### 2.3.1. Immunohistochemistry

Mice were anesthetized using diethyl ether and then perfused transcardially with 4% PFA in PBS. Brain samples were fixed in 4% PFA in PBS overnight at 4°C. GFP-M mice used in this study were 4–6 weeks of age. After dehydration in 20% sucrose in PBS, samples were embedded in OCT compound (Sakura Finetek, Japan). Cryosections were cut at 14 *μ*m thickness. For immunostaining, sections were incubated with anti-pCRMP2(S522) antibody at 4°C overnight. After washing with PBS, the secondary antibody, AlexaFluor, was applied, and sections were mounted with Vectashield. All immunostaining images were captured with a confocal microscope (FV1000).

#### 2.3.2. Rapid Golgi Staining

Male CRMP2^KI/KI^ and CRMP2^+/+^ mice at P18 and at 5 weeks of age (*n* = 3 for each genotype and age) were used in this study. For modified Golgi-Cox staining, an FD Rapid GolgiStain kit was used (FD NeuroTechnologies, MD). Stained slices were sectioned at a thickness of 200 *μ*m. Pyramidal hippocampal CA1 neurons in each mouse were selected for the analysis as described in our previous work [12, 13]. Dendritic spines of CA1 pyramidal neurons were counted in 50 *μ*m segments of proximal branches of apical dendrites under a BX50 microscope (Olympus) with a UPlanSApo 40x (NA = 0.95) objective. In a typical experiment, more than 2000 spines were counted on more than 50 dendritic segments in 25 neurons. Average spine densities per 50 *μ*m dendritic segments were then calculated for each genotype group. Groups of spines were compared using Student's *t*-test.

## 3. Results

### 3.1. CRMP2 Is Phosphorylated in Dendritic Spines of Cultured Hippocampal Neurons

**Figure 1:**
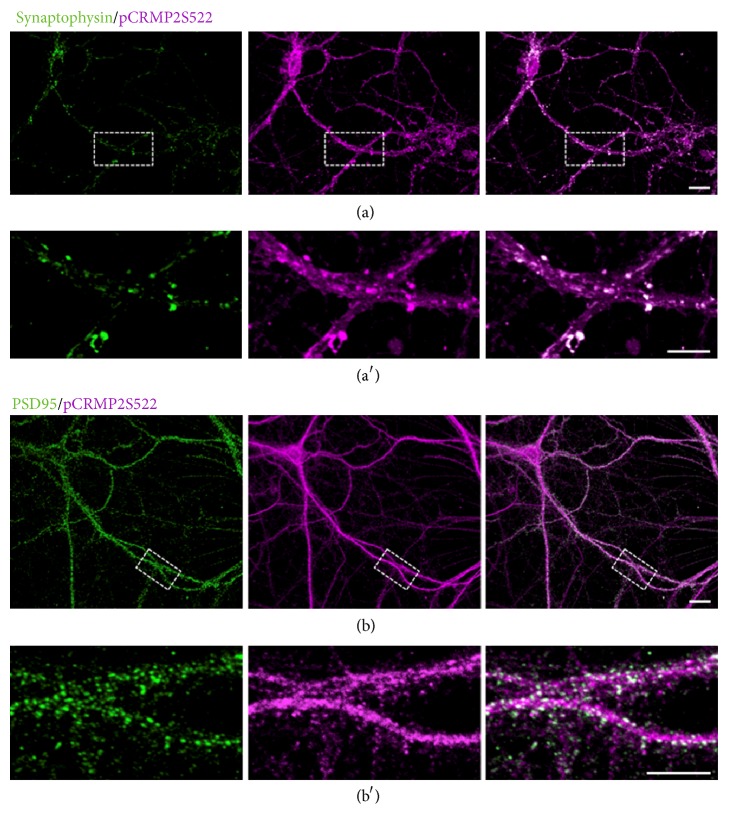
Subcellular localization of phospho-CRMP2 in cultured hippocampal neurons. (a) Immunocytochemistry with anti-phospho-CRMP2 and synaptophysin antibodies. Higher magnification is shown in (a′). (b) Immunocytochemistry with anti-phospho-CRMP2 and PSD95 antibodies in cultured rat hippocampal neurons 28 days* in vitro* (DIV). Merged images are shown. Highermagnification shown in (b′). Scale bar, 20 *μ*m.

We tested the possible function of CRMP2 phosphorylation in synapses. We first examined the subcellular localization of phospho-CRMP2 (pCRMP2) in cultured hippocampal neurons. We used anti-synaptophysin or anti-PSD-95 antibodies as presynaptic and postsynaptic markers, respectively. Double staining with anti-pCRMP2 and anti-synaptophysin or anti-PSD-95 antibodies showed that pCRMP2 colocalized with both synaptophysin and PSD-95 in dendritic protrusions (Figure 1). These results demonstrate that CRMP2 is phosphorylated by Cdk5 in the presynapse and dendritic spines in cultured hippocampal neurons, suggesting the possible involvement of CRMP2 phosphorylation in the development of dendritic spines in hippocampal neurons.

### 3.2. Reduced Spine Densities of Hippocampal CA1 Pyramidal Neurons in Juvenile CRMP2^KI/KI^ Mice

**Figure 2:**
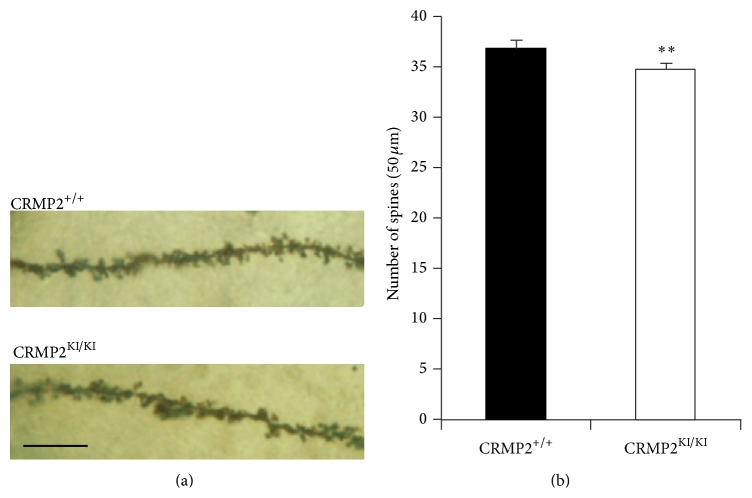
Reduction of dendritic spine density in hippocampal CA1 pyramidal neurons in CRMP2^KI/KI^ mice at P18. (a) Representative photographs of dendritic segments of hippocampal CA1 pyramidal neurons at P18. Scale bar, 10 *μ*m. (b) Reduced dendritic spine density was observed in hippocampal CA1 pyramidal neurons of CRMP2^KI/KI^ mice compared to those of control mice. 50 neurons in each area from three mice in each genotype were analyzed. ^*∗*^ *P* < 0.05.

We examined dendritic spine density by Golgi staining in P18 CRMP2^KI/KI^ mice. Golgi staining of forebrain slices showed a reduction in the number of spines in hippocampal CA1 pyramidal neurons in CRMP2^KI/KI^ mice compared with those in CRMP2^+/+^ mice (Figure 2). These results indicate that phosphorylation of CRMP2 by Cdk5 is required for proper formation of dendritic spines in the mouse brain.

### 3.3. CRMP2 Is Phosphorylated in Dendritic Spines of Hippocampal CA1 Pyramidal Neurons in Mouse Brains

**Figure 3:**
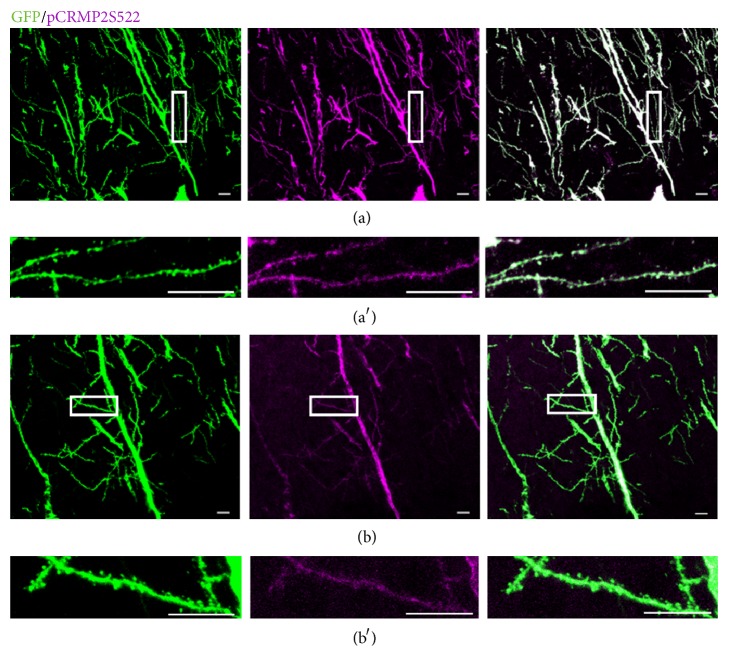
Localization of phospho-CRMP2 at dendritic spines of hippocampal CA1 pyramidal neurons. (a) Representative images of immunostaining of apical dendrites and their branches with phospho-CRMP2(S522) (pCRMP2S522) antibody in hippocampal CA1 pyramidal neurons from GFP-M mice. Magnified images of the areas indicated in (a) are shown in (a′). Scale bar, 10 *μ*m. (b) Representative images of immunostaining of apical dendrites and their branches with pCRMP2S522 antibody of hippocampal CA1 pyramidal neurons in GFP-M, CRMP2^KI/KI^ mice. Magnified images of the areas indicated in (b) are shown in (b′). Scale bar, 10 *μ*m.

CRMP2 is expressed in hippocampal neurons in adult mice [2]. Thus, we examined its phosphorylation in dendritic spines in hippocampal CA1 pyramidal neurons. For this purpose, we performed immunostaining of hippocampal sections from GFP-M mice at 4–6 weeks of age with anti-pCRMP2 antibody. In GFP-M mice, some hippocampal CA1 pyramidal neurons express GFP [15]. As shown in Figure 3, we detected pCRMP2 immunoreactivity in dendritic spines in hippocampal CA1 pyramidal neurons of GFP-M mice. In contrast its immunoreactivity was very low in those of GFP-M, CRMP2^KI/KI^ double mutant, which is attributable to a cross reactivity of this antibody to pCRMP1 [14]. These results suggest that Cdk5 phosphorylates CRMP2 in dendritic spines of hippocampal CA1 pyramidal neurons in the mouse brain.

### 3.4. Reduced Spine Densities of Hippocampal CA1 Pyramidal Neurons in 5-Week-Old CRMP2^KI/KI^ Mice

**Figure 4:**
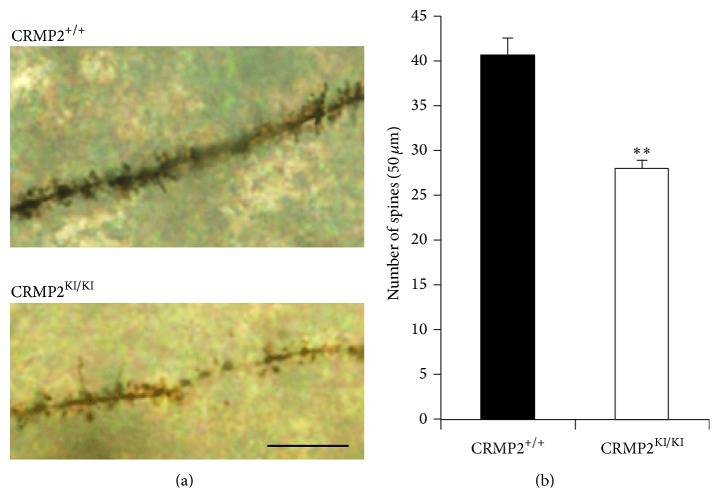
Reduction of dendritic spine density in hippocampal CA1 pyramidal neurons in 5 week-old CRMP2^KI/KI^ mice. (a) Representative photographs of dendritic segments of hippocampal CA1 pyramidal neurons at 5 weeks old. Scale bar, 10 *μ*m. (b) Reduced dendritic spine density was observed in hippocampal CA1 pyramidal neurons of CRMP2^KI/KI^ mice compared to those of control mice. 50 neurons in each area from three mice in each genotype were analyzed. ^*∗∗*^ *P* < 0.01.

We examined dendritic spine density by Golgi staining in 5-week-old CRMP2^KI/KI^ mice. Golgi staining of forebrain slices showed a reduction in the numbers of spines in hippocampal CA1 pyramidal neurons in CRMP2^KI/KI^ mice compared with those in CRMP2^+/+^ mice (Figure 4). These results indicate that phosphorylation of CRMP2 by Cdk5 is required for the development of proper dendritic spine density in the adult mouse brain.

## 4. Discussion

Recent studies have demonstrated that Cdk5 substrates are involved in the regulation of spine formation. Synaptic proteins phosphorylated by Cdk5 including ephexin1 [18], WAVE1 [19], CRMP1 [13], TrkB [20], PSD95 [21], drebrin [22], and p70 ribosomal S6 kinase (S6K) [23] have been shown to play a role in spine formation [13, 19, 21, 23] and maintenance [18, 20]. However, their functions differ such that some of them are crucial for spine formation [13] and some for spine retraction [18]. We recently reported reductions of dendritic spine densities in hippocampal CA1 pyramidal neurons of inducible-*p35* cKO,* p39* KO mice and CA1-*p35* cKO,* p39* KO mice with a p35 deletion in the CA1 region of the hippocampus after P17 [12]. We also reported reduction of spine densities in cerebral layer V neurons and hippocampal CA1 pyramidal neurons in inducible-*p35* cKO,* p39* KO mice when we deleted the p35 gene in 4-month-old animals [12]. These findings indicate that spine formation and maintenance are dependent on Cdk5 kinase activity in the mouse brain.

Our previous study showed that Sema3A-induced spine development is mediated by phosphorylation of CRMP1 by Cdk5 [13]. Because CRMP1 and CRMP2 have functional similarities in brain development [14], we examined whether phosphorylation of CRMP2 by Cdk5 is also important for the development and maintenance of dendritic spines* in vivo*. Cdk5 specifically phosphorylates Ser residue of CRMP2 at 522 [10]. We previously generated CRMP2^KI/KI^ mice to study the function of Cdk5-mediated CRMP2 phosphorylation by replacing Ser at 522 to Ala [14]. Our present analysis of dendritic spine densities in hippocampal CA1 pyramidal neurons in CRMP2^KI/KI^ mice at P18 showed reduced spine densities in these neurons compared to those of controls (Figure 2). Along with our previous study on CRMP1KO mice [13], these results indicate that CRMPs are important substrates of Cdk5 for spine formation. The results obtained in the present study will provide a new insight into the regulatory mechanisms underlying the effect of Cdk5 on dendritic spine density.

Our analysis of 5-week-old CRMP2^KI/KI^ mice showed further reduction of spine densities in hippocampal CA1 pyramidal neurons (Figure 4). These results exclude the possibility that reduced spine densities of hippocampal neurons in CRMP2^KI/KI^ mice at P18 (Figure 2) are due to the delay of brain development. In the cerebral cortex of macaque monkeys and humans, the number of dendritic spines rapidly increases after birth and peaks in an early phase of the infantile period [24]. Spine density then decreases during the later infantile period and adolescence period to reach the adult level [25]. Decrease of dendritic spine density during the transition from puberty to adulthood has also been reported in the mouse hippocampus [26]. These studies indicate ontogenetic similarity between rodent, primate, and human in spine formation and pruning. This overshoot-type time course of spine formation and pruning is attractive for researchers because it is possibly involved in developmental and psychiatric disorders [27]. Further studies are also required for the analysis of the involvement of CRMP2 phosphorylation in spine pruning and maintenance.

Cdk5 and its activator p35 play multiple roles in brain development, especially in neuronal migration [28]. Emerging evidence suggests that Cdk5/p35 is also involved in synaptic plasticity [29]. Cdk5/p35 is localized at neuronal synapses and phosphorylates many synaptic proteins [21, 30–33]. Furthermore, the induction of synaptic plasticity and spatial learning are impaired in Cdk5/p35 mutant mice [34–36]. The role of Cdk5 in synaptic plasticity and learning was initially studied using Cdk5 inhibitors, which showed inhibition of hippocampal LTP induction and context-dependent fear conditioning [30, 37]. We have previously reported the impairment of long-term depression (LTD) induction and spatial learning and memory in p35 KO mice [36]. Our recent study of p35 conditional KO (cKO) mice, which lack histological abnormalities in the brain, also showed impairment of spatial learning and memory and LTD induction [38]. Importantly, electrophysiological analysis of hippocampal slices from p35 cKO mice revealed reduced synaptic transmission in hippocampal CA1 pyramidal neurons [38]. Since we observed reduced spine densities of hippocampal CA1 pyramidal neurons in CRMP2^KI/KI^ mice, further electrophysiological studies of hippocampal synaptic plasticity and behavioral analysis in CRMP2^KI/KI^ mice will provide further knowledge of the significance of Cdk5-mediated CRMP2 phosphorylation in synaptic plasticity and in learning and memory.

## 5. Conclusions

CRMP2 is phosphorylated in dendritic spines of rodent hippocampal neurons* in vitro *and* in vivo*. When we eliminated Cdk5-mediated phosphorylation of CRMP2 at S522 in the mouse brain, the densities of dendritic spines of hippocampal neurons were reduced in the mouse brain. These results suggest the regulation of spine density of hippocampal neurons by CRMP2 phosphorylation.
